# Bilioenteric bypass stricture type II with hepatolithiasis: A case report

**DOI:** 10.1016/j.amsu.2020.07.011

**Published:** 2020-07-11

**Authors:** Warsinggih, Amir Fajar, Ibrahim Labeda, Julianus Aboyaman Uwuratuw, Prihantono, Muhammad Faruk

**Affiliations:** a Division of Digestive Surgery, Department of Surgery, Faculty of Medicine, Hasanuddin University, Makassar, Indonesia; b Department of Surgery, Faculty of Medicine, Hasanuddin University Makassar, Indonesia

**Keywords:** Roux-en-Y Reconstruction, Anastomotic biliary strictures, Bile duct, Case report

## Abstract

**Introduction:**

Secondary hepatolithiasis can occur as a result of bilioenteric stenosis or biliary anastomosis stenosis. The incidence of secondary hepatolithiasis appears to increase with increasing rates of hepatobiliary surgery. Here we report the first reported case of secondary hepatolithiasis.

**Case presentation:**

A 57-year-old female patient complaining of jaundice all over the body since two years ago. The jaundice was intermittent and progressive. There was a history of previous bilioenteric bypass hepaticojejunostomy Roux-en-Y due to common bile duct cyst. On investigation, we found obstructive jaundice due to stricture of bilioenteric anastomosis type II after bilioenteric bypass hepaticojejunostomy Roux-en-Y with hepatolithiasis type II LR, according to the Takada classification. We did laparotomy found bilateral hepatic duct dilatation, we make incision and remove multiple stones. And then, we performed choledochoscope and confirm total occlusion of tract to distal common hepatic duct. We performed reconstruction Roux-en-Y hepaticojejunostomy with stenting. During the follow-up period, our patients were disease-free.

**Conclusion:**

Stricture of bilioenteric anastomosis were successfully treated by surgical reconstruction Roux-en-Y hepaticojejunostomy and stenting. This management has a good outcome and could be an effective alternative to surgery.

## Highlights

• Secondary hepatolithiasis can occur as a result of bilioenteric stenosis or biliary anastomosis stenosis.
• Here we report the first reported case of bilioenteric bypass stricture type II with Hepatolithiasis.
• Combination surgical reconstruction Roux-en-Y hepaticojejunostomy and stenting has a good outcome.

## 1 Introduction

Surgery involving the hepatobiliary system, as in the case of choledochal cysts, can increase the risk of secondary hepatolithiasis [1]. Bilioenteric bypass procedures e.g., Roux-en-Y hepaticojejunostomy used in such cases, can cause complications such as biliary stricture at the site of the anastomosis. Bilioenteric stenosis or biliary anastomosis stenosis can cause secondary hepatolithiasis that results in obstructive jaundice [2]. The incidence of secondary hepatolithiasis appears to increase with increasing rates of hepatobiliary surgery and life expectancy [1]. Although usually benign, hepatolithiasis is associated with recurrent biliary stricture, cholangitis, hepatic abscess, hepatic atrophy, hepatic cirrhosis, and poor prognostic factors in intrahepatic cholangiocarcinoma [3]. To the best of our knowledge, and this is the first reported case of secondary hepatolithiasis. We reported the case in accordance to the SCARE 2018 guidelines [4].

## 2 Case report

A 57-year-old woman was admitted with jaundice all over the body in the last two years. The jaundice was intermittent and progressive. This complaint was accompanied by weight loss of around 20 kg in the last one year. There was no nausea, vomiting, abdominal pain, itching on the body, and fever. The patient reported dark urine, and pale stools manifested in the last six months. There was a history bilioenteric bypass hepaticojejunostomy Roux-en-Y due to common bile duct cyst, two years previously. There was no history of chronic disease. There was no family history of jaundice. Vital sign within normal limits.

**Fig. 1:**
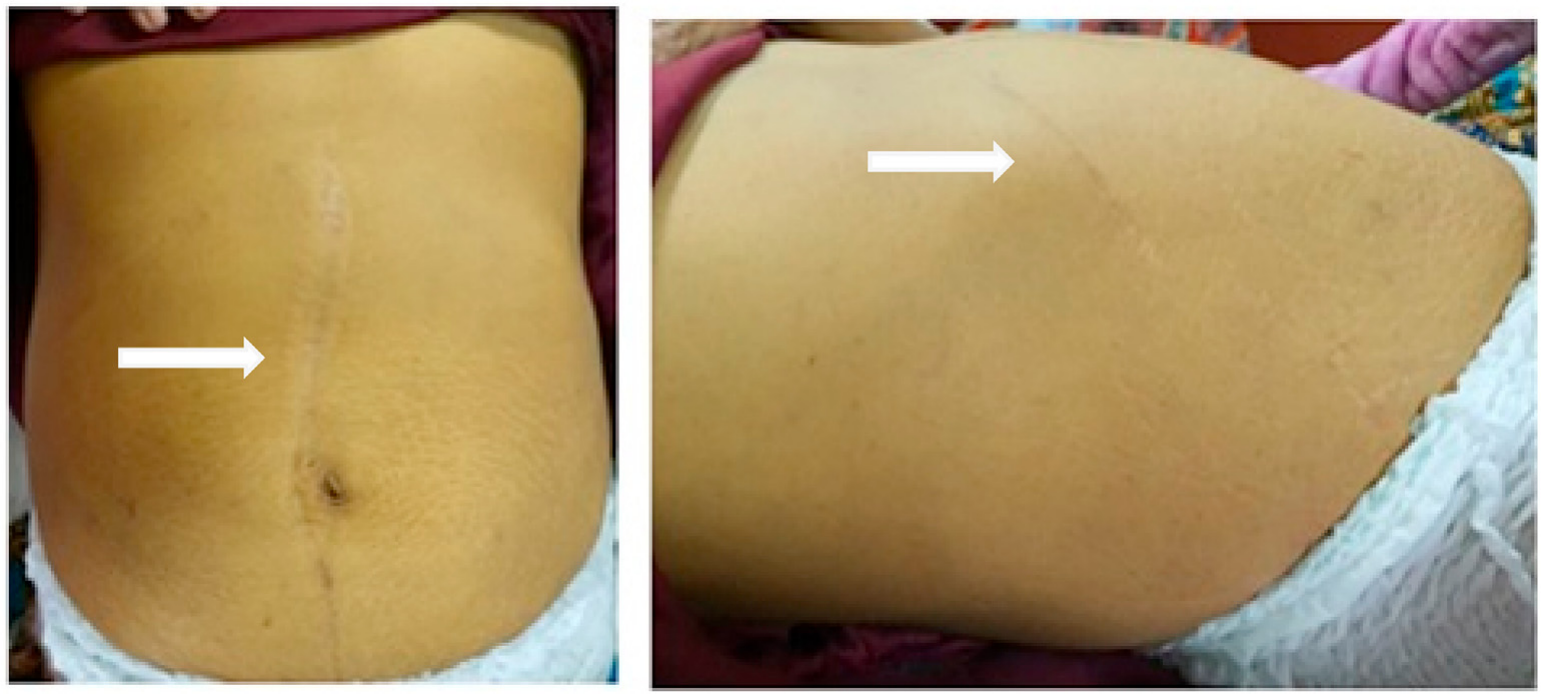
Jaundice and surgery scar on inspection of the abdomen (white arrows).

**Fig. 2:**
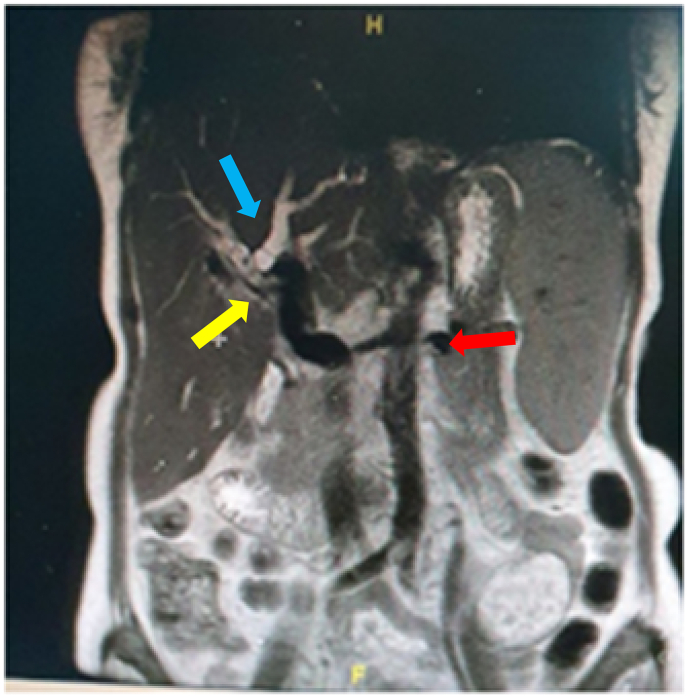
Non-Contrast MRCP showed dilated right and left intrahepatic duct containing multiple stones. (blue arrow), stricture of bilioenteric anastomosis (yellow arrow), bilioenteric bypass hepaticojejunostomy Roux-en-Y (red arrow) and hepatosplenomegaly. (For interpretation of the references to colour in this figure legend, the reader is referred to the Web version of this article.)

Physical examination revealed a lack of nutritional status, the presence of anemic conjunctiva, scleral icterus, jaundice, and surgical scars in the abdomen (Fig. 1). On rectal toucher examination, a pale stool was found. From laboratory tests found a decrease in hemoglobin (7.6 mg/dl), negative results for serum HBsAg tests, and an increase in liver enzymes (AST 177 U/L and ALT 85 U/L), serum direct bilirubin 18.66 mg/dl, and total serum bilirubin level 20.15 mg/dl. Chest X-ray examination within normal limits. On non-contrast MRCP showed dilated right and left intrahepatic duct containing multiple stones, and stricture of bilioenteric anastomosis (Fig. 2). The patient was diagnosed with obstructive jaundice due to stricture of bilioenteric anastomosis after bilioenteric bypass hepaticojejunostomy Roux-en-Y with hepatolithiasis type II LR according to the Takada classification.

**Fig. 3:**
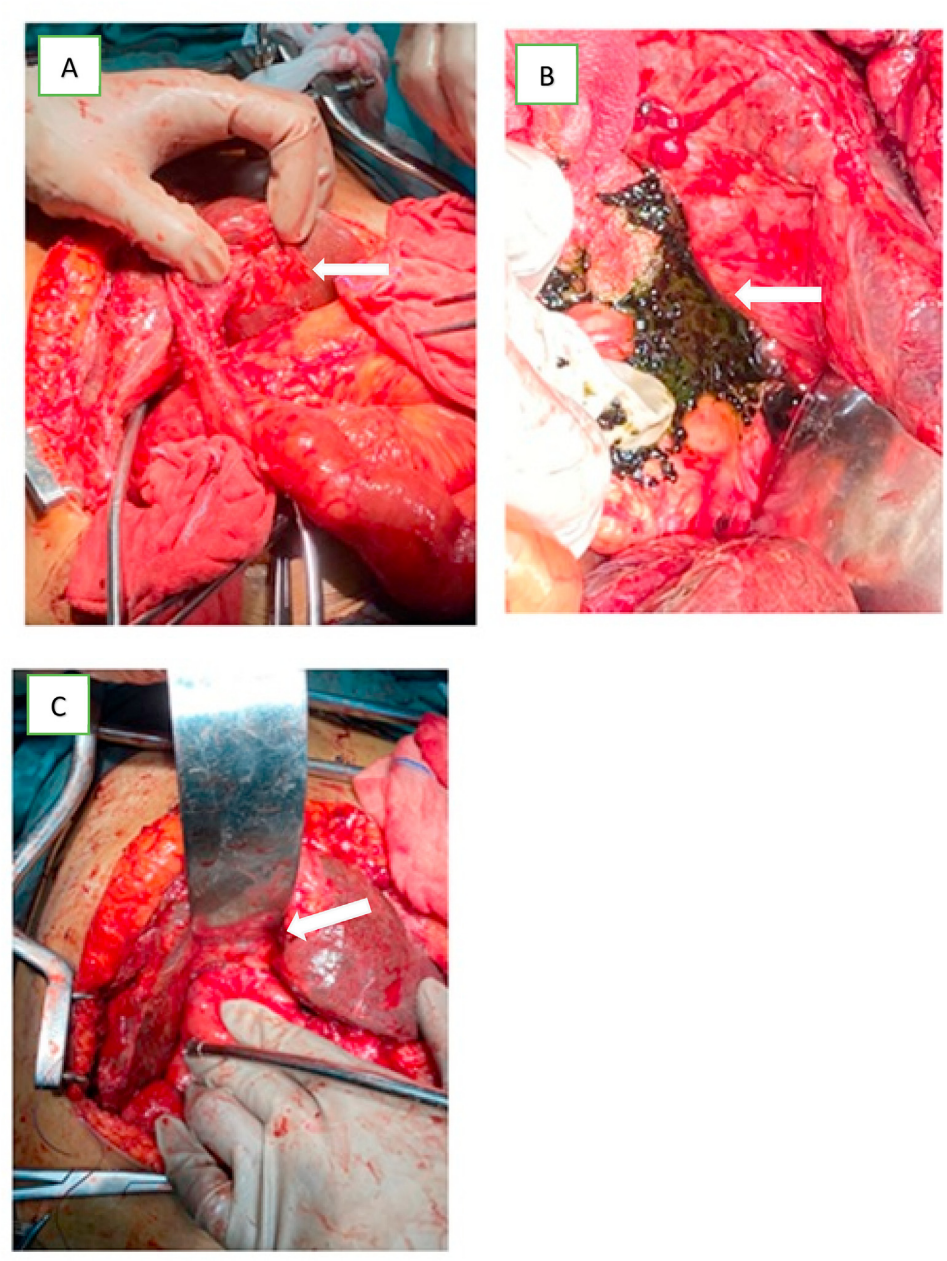
Laparotomy bilioenteric anastomosis reconstruction: A) stricture of bilioenteric anastomosis (arrow); B) hepatolithiasis multiple with sludge (arrow); C) reconstruction Roux-en-Y hepaticojejunostomy (arrow).

Exploratory laparotomy bilioenteric anastomosis reconstruction was then we decided to do (Fig. 3). During surgery, a bilateral subcostal incision (Mercedes incision) and adhesiolysis was performed. We found bilateral hepatic duct dilatation, we make an incision and remove multiple stones. And then, we performed a choledochoscope and confirmed the total occlusion of the tract to the distal common hepatic duct. We did reconstruction Roux-en-Y hepaticojejunostomy with stenting from the left hepatic duct to the right flank.

The postoperative management includes oral feeding administered via nasogastric tube after 48 h of aspiration, H2 blockers, and antibiotics drug, monitoring of complete blood count, and hepatic function for the first four postoperative days. After discharge, clinical conditions and liver function are monitored at 1, 3, and 6 months from surgery. Liver morphological findings were assessed by assessed radiography such as abdominal ultrasonography (US) and MRCP. During the follow-up period, our patients were disease-free.

## 3 Discussion

Obstructive jaundice is a condition where there is a partial or complete blockage of the flow of bile and its components into the intestinal tract. Cholestasis classified into intrahepatic cholestasis, and extrahepatic cholestasis [5]. Hepatolithiasis is one of the causes of obstructive jaundice. Hepatolithiasis is associated with recurrent cholangitis, biliary stricture, hepatic abscess, liver atrophy, liver cirrhosis, and poor prognostic in intrahepatic cholangiocarcinoma [1]. The incidence of this disease (hepatolithiasis) varies, but it is quite common in East Asia, with an incidence rate of around 2–25%. In the Western world, hepatolithiasis is rare, with incidents reported between 0.6% and 1.3% [3].

Secondary hepatolithiasis can occur as a result of bilioenteric stenosis or biliary anastomosis stenosis [2]. The incidence of secondary hepatolithiasis appears to increase with increasing rates of hepatobiliary surgery and life expectancy [1]. Catena et al. reported an incidence secondary hepatolithiasis due to biliary injury due to cholecystectomy was about 35.2% [6]. Nowadays, the incidence of bile duct injury due to laparoscopic cholecystectomy near to 0.6% [2].

In this case, reported the most likely cause is stricture of bilioenteric anastomosis after bilioenteric bypass hepaticojejunostomy Roux-en-Y due to common bile duct cyst. Secondary hepatolithiasis that results in biliary obstruction has clinical manifestations that are similar to primary strictures. Symptoms that can appear are jaundice, fatigue, fever, abdominal pain, gastrointestinal bleeding, increased liver function, and hyperbilirubinemia [2,5].

Abdominal ultrasonography (USG) and computed tomography (CT) scans are the primary imaging modalities for hepatolithiasis. Magnetic resonance imaging (MRI) and magnetic resonance cholangiopancreatography (MRCP) can provide more explicit images of the bile duct and can detect stones without exposing the patient to radiation [3].

Classification of hepatolithiasis according to Takada and colleagues. It is divided into five types according to the location of stones and strictures. Type I: no strictures in intrahepatic and extrahepatic biliary tract, with mild dilation of the biliary system. Type II: biliary stricture exists in the lower bile duct or ampulla of the duodenum, showing remarkable dilation on the bile ducts. Type III: stricture at the hepatic hilum. Type IV: biliary stricture in the unilateral hepatic lobe. Type V: multiple biliary strictures in the bilateral hepatic lobe or bilateral congenital biliary cysts [1]. Patients with stones both in the intrahepatic and extrahepatic bile duct as being type IE. In addition, patients were grouped by the location of stones as follows: left side: type L, right side: type R, right and left sides: type LR, and caudate lobe: type C. In this case, considering the symptoms, physical examination, and radiological examination, the most likely diagnosis was obstructive jaundice due to stricture of bilioenteric anastomosis after bilioenteric bypass hepaticojejunostomy Roux-en-Y with hepatolithiasis type II LR according to the Takada classification.

Meanwhile Bismuth classified biliary strictures according to the distance from the hilar structure (Table 1) [7]. In our cases, patient was diagnosed with obstructive jaundice due to stricture of bilioenteric anastomosis type II after bilioenteric bypass hepaticojejunostomy Roux-en-Y with hepatolithiasis (according to Bismuth classification).

**Table 1:** Bismuth classification of biliary structures.

Type	Definition
I	Common hepatic or main bile duct stump ≥2 cm
II	Common hepatic duct stump <2 cm
III	Ceiling of the biliary confluence is intact; right and left ductal systems communicate
IV	Ceiling of the confluence is destroyed; bile ducts are separated
V	Involves the aberrant right sectoral hepatic duct alone or with concomitant injury of CHD

The management of hepatolithiasis is complex, requiring a multidisciplinary treatment with the aim of removing stones and bile stasis. Hepatolithiasis management methods include non-surgical treatment (percutaneous transhepatic cholangioscopy lithotripsy) and surgery such as hepatectomy [3,8,9]. Surgery involving the hepatobiliary system as in the case of choledochal cysts with cholecystectomy surgery can increase the risk of secondary hepatolithiasis [1,2]. Bilioenteric bypass procedures e.g., Roux-en-Y hepaticojejunostomy, are the most frequent procedures and give the best outcome in terms of outcomes for revision surgery [10].

Revision surgery is the first-choice treatment for recurrent biliary stricture after reconstructive surgery. The Hepp-Couinaud will be the most adequate approach choice for this surgery. The extrahepatic main left duct can be easily resolved with this technique [2,11].

An alternative to surgery is the placement of a stent with balloon dilation through a percutaneous or endoscopic approach [12]. Benign anastomosis stricture after the bilioenteric bypass procedure raises difficult management problems. Therapeutic options include reconstruction or surgeries with a minimally invasive approach [2,13]. As in this case, the patient underwent laparotomy adhesiolysis found dilatation bilateral hepatic duck. A reconstruction Roux-en-Y hepaticojejunostomy procedure was performed to repair biliary strictures and remove the stone, and we combined this procedure with stenting by large external-internal drainage (14 Fr), for more than four weeks to drainage of the stones that were likely still remaining and reduce the risk of stricture relapse.

One of the main complications of this Roux-en-Y technique, although uncommon, is that biliary stricture can exist at the site of anastomosis as an outcome of fibrotic healing. Post-op stricture formation at the anastomotic site varies throughout the literature from 4 to 38% [14]. Younger age was associated with a decreased likelihood of stricture formation. A more significant proportion of patients who underwent an operation for hepatolithiasis due to benign biliary strictures (BBS) were more likely to develop stricture because patients with malignant obstructions due to malignancy have dilated ducts reducing the likelihood of stricture development. Patients with malignancy disease have poor survival rates, which likely die before this complication can develop [2,14].

Although surgical reconstruction with Hepp-Couinaud approach is considered the most definitive treatment, the morbidity rate is around 25%, with a reported mortality rate of 2%–13% [2,10,13] and a success rate was 97% and 94% [2]. The majority of patients with failure of biliary repair will begin to develop symptoms within 5–7 years postoperatively. So prolonged follow-up is necessary [2,11,15]. Patients must be routinely controlled 2–4 times during the first year after revision surgery. Evaluation should include clinical examination, liver function tests (LFT), abdominal ultrasound (US), if necessary, with abdominal CT scan. After that, follow-up can be done one to two times per year for a minimum of 5 years [2].

## 4 Conclusion

Stricture of bilioenteric anastomosis were successfully treated by surgical reconstruction Roux-en-Y hepaticojejunostomy and stenting. This management has good outcomes and could be an effective alternative to surgery.

## Provenance and peer review

Not commissioned, exteranlly peer reviewed.

## Ethical approval

The study is exempt from ethical approval in our institution.

## Please state any sources of funding for your research

No funding or sponsorship.

## Author contribution

WS, AF, IL, JAB, PRI and MF researched the literature and wrote the manuscript. WS, AF, and MF operated on the patient and had the idea for this case report. WS, AF, IL, JAB, and PRI checked the manuscript and made corrections. WS, PRI, and MF provided the overall guidance and support. All authors read and approved the final manuscript.

## Please state any conflicts of interest

The authors declare that they have no conflict of interests.

## Registration of research studies

None.

## Guarantor

Warsinggih.

## Consent

Written informed consent was obtained from the patient for publication of this case report and accompanying images. A copy of the written consent is available for review by the Editor-in-Chief of this journal on request.

## Declaration of competing interest

The authors declare that they have no conflict of interests.
